# Stimulation of zona incerta selectively modulates pain in humans

**DOI:** 10.1038/s41598-021-87873-w

**Published:** 2021-04-26

**Authors:** Charles W. Lu, Daniel E. Harper, Asra Askari, Matthew S. Willsey, Philip P. Vu, Andrew D. Schrepf, Steven E. Harte, Parag G. Patil

**Affiliations:** 1 Department of Neurosurgery, University of Michigan, 1500 E Medical Center Drive, SPC 5338, Ann Arbor, MI 48109-5338 USA; 2 Department of Biomedical Engineering, University of Michigan, Ann Arbor, MI USA; 3 Department of Anesthesiology, Emory University, Atlanta, GA USA; 4 Department of Anesthesiology, Chronic Pain and Fatigue Research Center, University of Michigan Medical School, Ann Arbor, MI USA; 5 Division of Rheumatology, Department of Internal Medicine, University of Michigan Medical School, Ann Arbor, MI USA

**Keywords:** Sensory processing, Neurological disorders

## Abstract

Stimulation of zona incerta in rodent models has been shown to modulate behavioral reactions to noxious stimuli. Sensory changes observed in Parkinsonian patients with subthalamic deep brain stimulation suggest that this effect is translatable to humans. Here, we utilized the serendipitous placement of subthalamic deep brain stimulation leads in 6 + 5 Parkinsonian patients to directly investigate the effects of zona incerta stimulation on human pain perception. We found that stimulation at 20 Hz, the physiological firing frequency of zona incerta, reduces experimental heat pain by a modest but significant amount, achieving a 30% reduction in one fifth of implants. Stimulation at higher frequencies did not modulate heat pain. Modulation was selective for heat pain and was not observed for warmth perception or pressure pain. These findings provide a mechanistic explanation of sensory changes seen in subthalamic deep brain stimulation patients and identify zona incerta as a potential target for neuromodulation of pain.

## Introduction

Deep brain stimulation (DBS) has been used for treatment of pain since the 1970s. Stimulation of classical targets—sensory thalamus, periaqueductal gray, and periventricular gray matter—is often able to provide pain relief, although long-term success of these interventions varies widely across etiologies. DBS treatment of phantom limb pain, failed back surgery syndrome, and trigeminal neuropathy is frequently successful; however, outcomes for other etiologies, including stroke, peripheral neuropathy, and brachial plexus injury, tend to be less satisfactory^1–3^. The many pain patients for whom conventional DBS remains ineffective highlight the need for new targets of neuromodulation.

**Figure 1:**
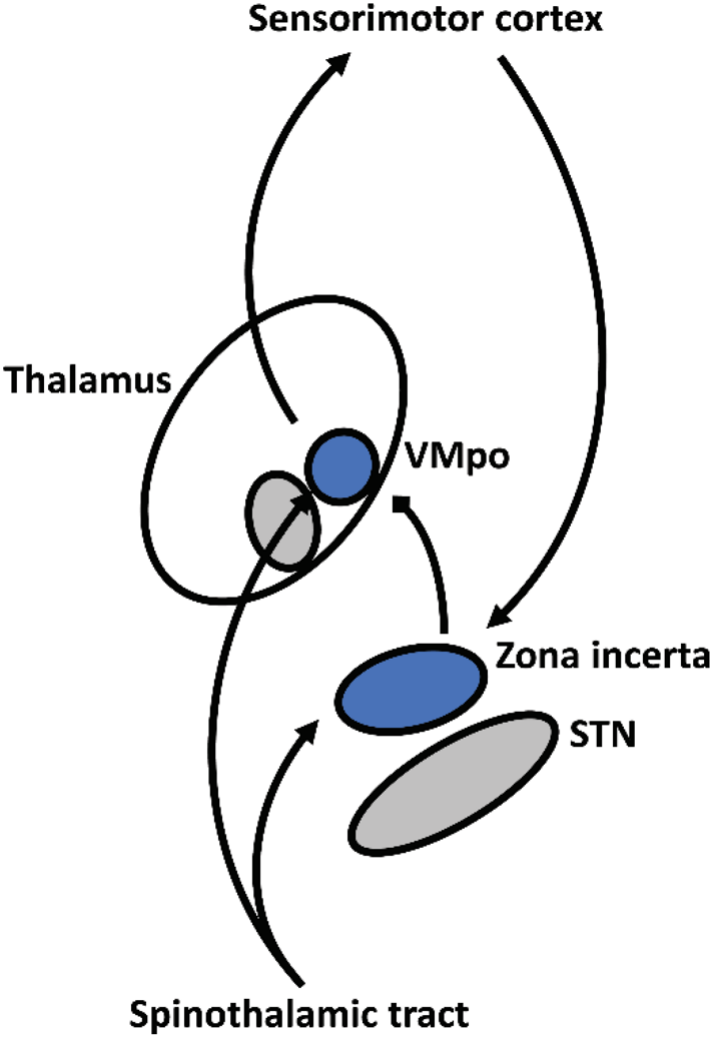
Feedforward inhibition of thalamic pain processing by zona incerta. *VMpo* ventromedial posterior nucleus.

Emerging evidence points to zona incerta, a heterogeneous region of cell bodies and fibers dorsal to subthalamic nucleus, as a promising new target for pain neuromodulation. The region receives direct spinothalamic input and projects GABAergic efferents to ventromedial thalamus, which integrates cortical and spinothalamic inputs (Fig. 1)^4, 5^. In a rodent model of central pain, zona incerta was shown to act as a feedforward inhibitor of pain perception^6^. The same group later demonstrated that 50–60 Hz stimulation of zona incerta reduces hyperalgesia in a rat model of neuropathic pain, providing a proof of concept for analgesic zona incerta DBS^7^. More recent work has established compelling causative links between GABAergic output from zona incerta, neuropathic pain, and neuromodulatory relief of hyperalgesia and allodynia^8–10^.

Experimental findings in rodent models have been corroborated by observations of sensory changes in human patients receiving subthalamic DBS. Although subthalamic DBS for Parkinson disease nominally targets the subthalamic nucleus, a large body of work has shown that most active contacts are located at or above the dorsal border of the subthalamic nucleus—a region directly adjacent to and overlapping zona incerta^11, 12^. While the intervention is best recognized for its suppression of parkinsonian motor symptoms, it is also known to have substantial therapeutic effects on pain and sensation^13–17^. Multiple studies show that this effect is not explained by motor improvements alone^18, 19^, indicating an independent mechanism by which subthalamic stimulation ameliorates pain symptoms. Taken together, these observations strongly suggest that stimulation of zona incerta modulates pain perception.

In this study, we utilized the serendipitous placement of subthalamic DBS leads in patients with Parkinson disease to directly evaluate the effects of zona incerta DBS on human perception of experimental heat and mechanical pain. A broad set of stimulation parameters were tested and then prospectively validated in independent cohorts, showing that stimulation at physiological frequency modulated perceived heat pain by a modest but significant amount. Modulation was specific to heat pain and did not significantly alter perception of non-painful heat or mechanical pain. These findings provide a mechanistic explanation of sensory changes seen in subthalamic DBS patients and identify zona incerta as a potential target for neuromodulation of pain.

## Results

### Subjects

Subjects scored an average of 3.1 points (standard deviation of 2.5 points) on the Geriatric Depression Scale Short Form, with one subject (at 11 points) exceeding the 10-point cutoff for depression risk (included in analysis) (Table 1). One subject was a non-responder to hot stimuli up to 45 °C and was excluded from all analyses. Results were collected unilaterally on one subject due to scarring on one arm from previous traumatic injury. Unilateral data from one subject was excluded due to misinterpretation of subject instructions. No subjects reported pre-existing pain in the areas examined in this study.

**Table 1:** Subject characteristics.

Age	Sex	GDSS	Duration of DBS, months	Experiment	Data inclusion
62	M	4	55	Exploratory	Bilateral
58	M	2	18	Exploratory	Bilateral
62	M	2	64	Exploratory	Excluded
67	M	2	13	Exploratory	Bilateral
58	F	1	65	Exploratory	Bilateral
71	M	2	59	Exploratory	Bilateral
73	M	2	7	Exploratory	Unilateral
45	M	1	22	Validation	Bilateral
62	F	11	6	Validation	Bilateral
60	M	3	40	Validation	Bilateral
73	F	2	42	Validation	Bilateral
66	M	2	38	Validation	Unilateral

*GDSS* Geriatric Depression Scale Short Form score. Duration of DBS measured from time of implant to time of study.

### Zona incerta DBS modulates heat pain

**Figure 2:**
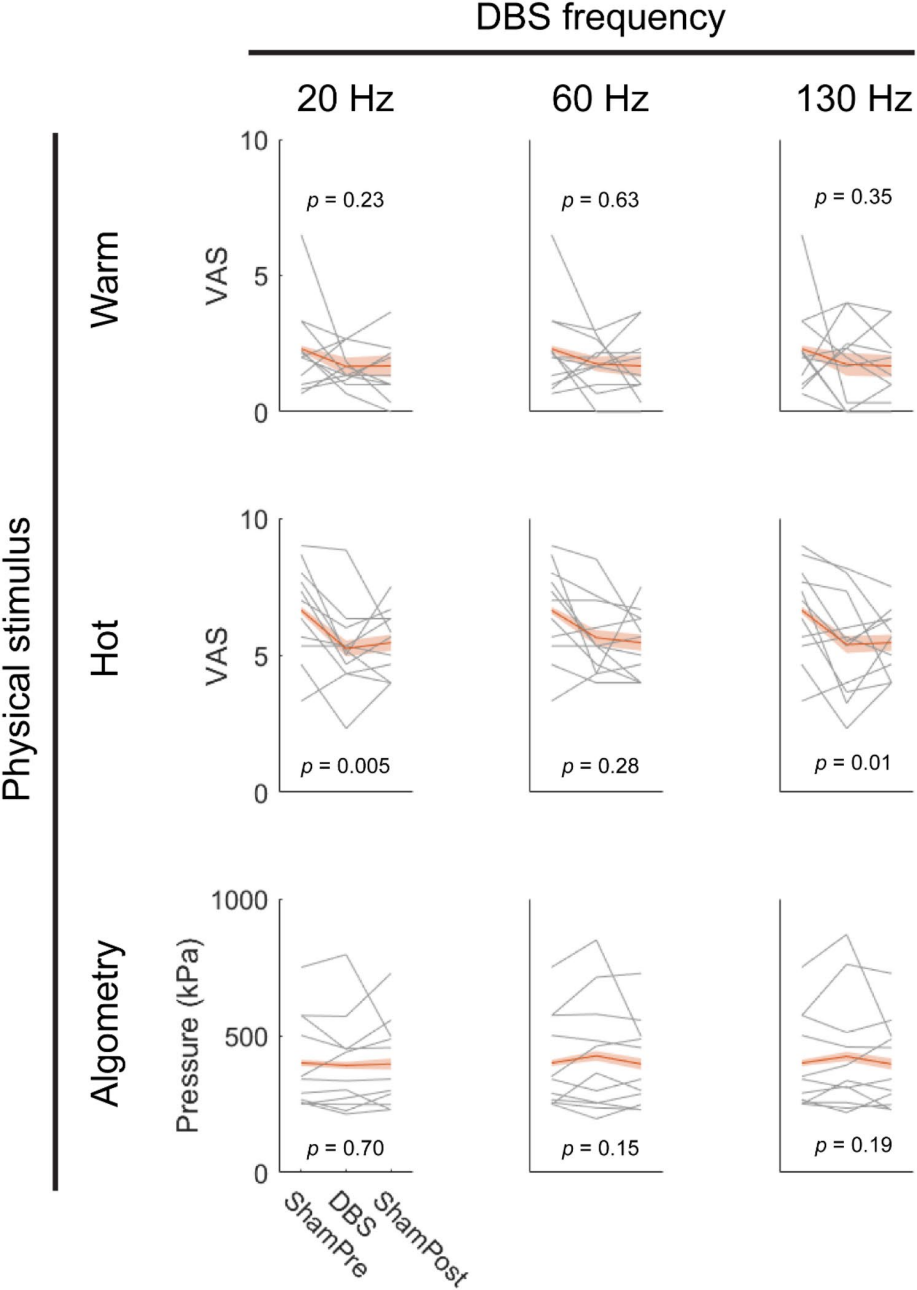
Effects of DBS on perceived pain from warm stimuli, hot stimuli, and mechanical pressure. Gray lines show mean pain scores across arm sites for each subject-implant. Red lines show average across implants with standard error of the mean shaded. Note that intra-subject changes tend to occur across a small range within the visual-analog scale, provided in full for data transparency. *n* = 99 trials for all analyses shown, with sham trials shared across DBS frequencies. *VAS* visual analog scale.

Zona incerta DBS with conventional 130 Hz stimulation decreased perceived heat pain by 0.71 points [standard error (SE) = 0.28, *p* = 0.01, n = 99] on the visual analog scale (Fig. 2). Low frequency 20 Hz stimulation reflecting physiological firing of zona incerta also reduced pain elicited by hot stimuli (SE = 0.27, − 0.78 points; *p* = 0.005). DBS of any frequency did not appear to significantly affect perceived pain from warm stimuli (20 Hz, *p* = 0.23; 60 Hz, *p* = 0.63; 130 Hz, *p* = 0.35; n = 99 for all comparisons) or mechanical pain thresholds (20 Hz, *p* = 0.70; 60 Hz, *p* = 0.15; 130 Hz, *p* = 0.19; n = 99 for all comparisons).

### Validation of 20 and 130 Hz stimulation

**Figure 3:**
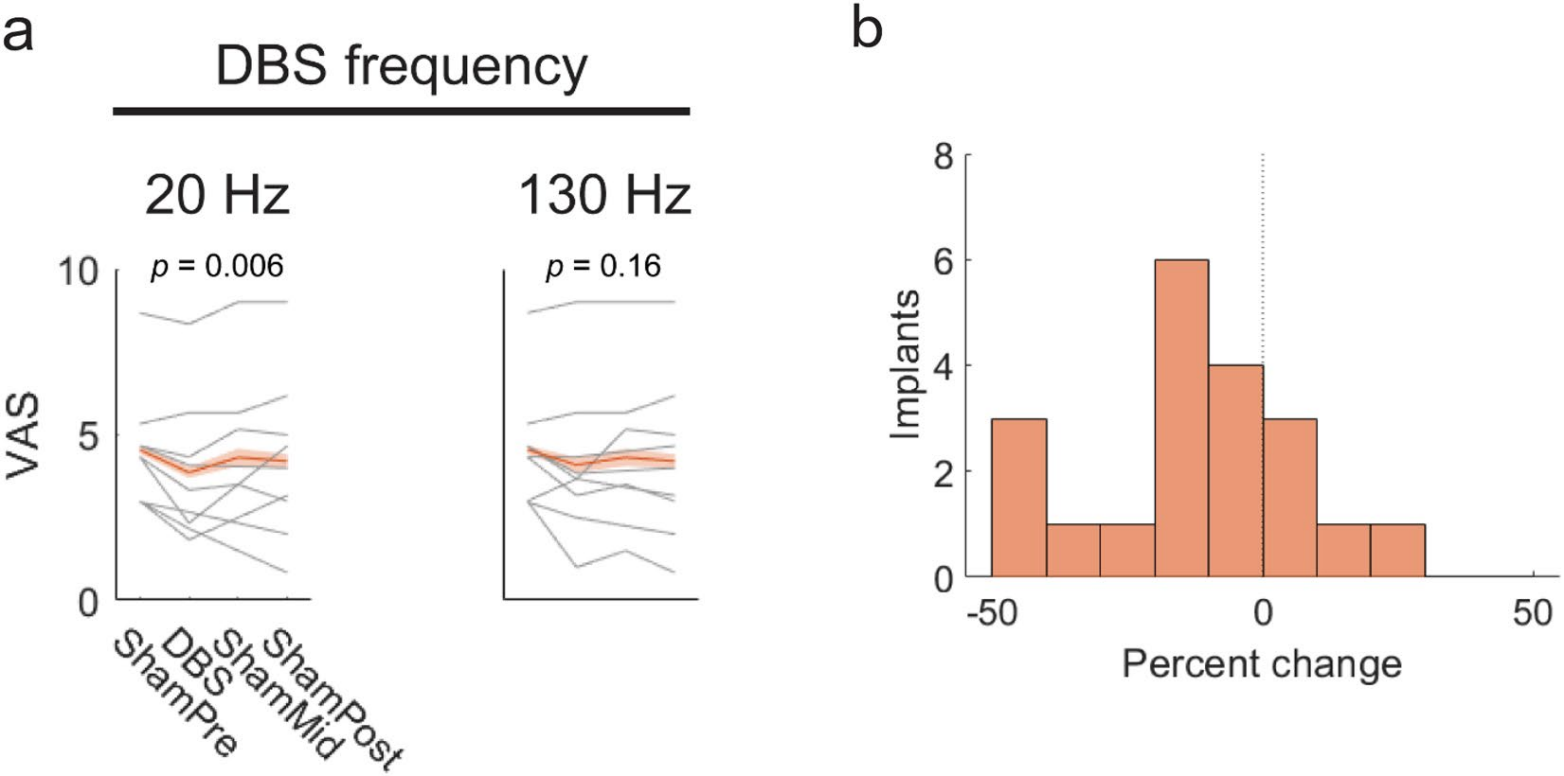
Effects of 20 and 130 Hz DBS on perception of heat pain. (**a**) Effects of DBS on perceived pain from hot stimuli. Gray lines show mean pain scores across arm sites for each subject-implant. Red lines show average across implants with standard error of the mean shaded. *n* = 96 trials for both analyses, with sham trials shared across DBS frequencies. (**b**) Distribution of percent change in heat pain with 20 Hz DBS. *n* = 20 implants.

Due to the small effect sizes observed and multiple comparisons made in the exploratory experiments, the effects of 20 and 130 Hz stimulation on heat pain were measured in an independent set of nine implants (five subjects) to confirm results. This group also received an additional sham trial. In this cohort, 20 Hz stimulation reduced heat pain by 0.51 points (SE = 0.18, *p* = 0.006, n = 96), confirming the original observation of this effect (Fig. 3a). Stimulation at 130 Hz also reduced heat pain by 0.27 points but did not reach significance (*p* = 0.16, n = 96). As the validation experiments did not incorporate wash-in time, results also indicate that neuromodulation of heat pain by zona incerta DBS takes rapid effect.

### Quantification of effect size and sites

**Figure 4:**
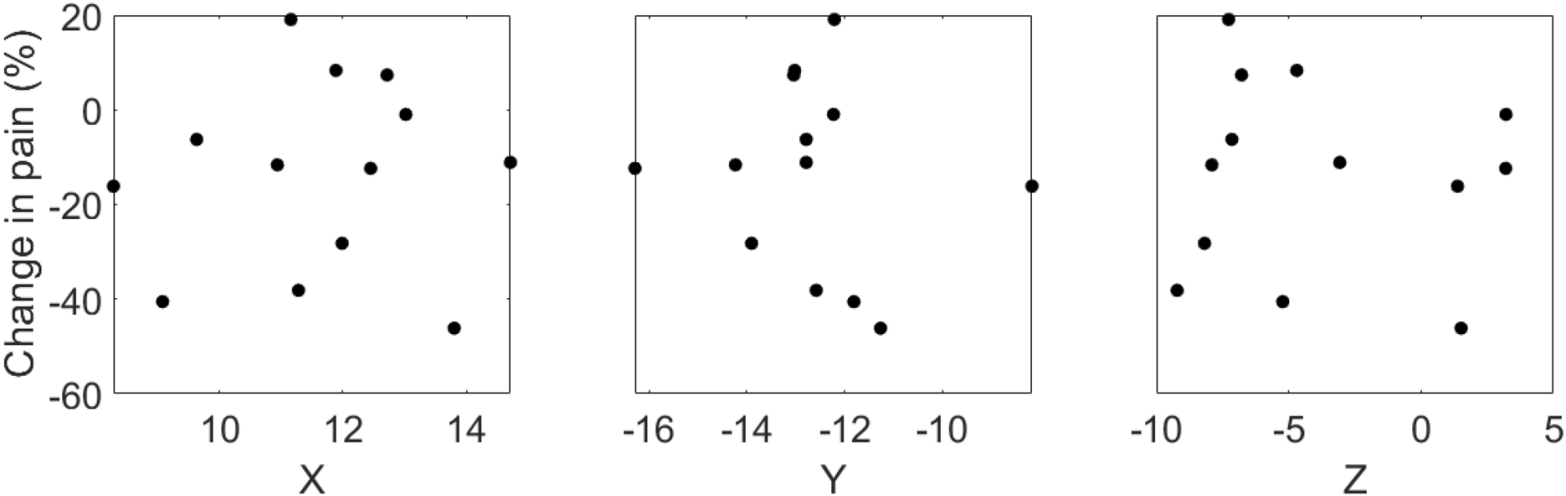
Percent change in pain from 20 Hz DBS plotted against active contact location in MNI coordinates (mm).

To quantify the effects of 20 Hz stimulation on heat pain, data from exploratory and validation experiments were combined. Analysis of individual subject-implant changes from individually averaged sham scores revealed analgesic effect at 10/13 implants, with pain reduction of 30% or more in 20% of implants. The mean and median effects of stimulation on heat pain were − 11.8% and − 11.3%, respectively, with a standard error of 4.5% (Fig. 3b). Analysis of stimulation sites did not reveal correlation between analgesic effect and the site of stimulation in MNI atlas space (Fig. 4). Locations of responder and non-responder stimulation sites are shown in MNI space in Supplementary Fig. S2.

## Discussion

This study evaluates a new form of DBS for pain and demonstrates that stimulation of zona incerta achieves a modest but significant analgesic effect in human subjects. We further demonstrate that analgesia is best achieved using stimulation at low frequency. This phenomenon was selective to perception of heat pain and did not affect perceived intensity of warm or mechanical stimuli. The results of this study are the first to confirm that stimulation of zona incerta modulates evoked pain perception in humans. This follows a compelling body of work in rodent models, which have demonstrated both behavioral manifestations and mechanistic explanations of pain modulation by excitation of zona incerta^7, 8^. Human translation of zona incerta DBS is an important step to better qualify the perceptual effects of zona incerta neuromodulation and lays a foundation for further optimization of analgesic DBS.

Observation that analgesia is best achieved with stimulation at physiological frequency is a notable finding. While stimulation at conventional DBS frequencies has been hypothesized to act as an informational lesion^20^, stimulation at physiological firing rates may act to increase activity in zona incerta, which has been shown by rodent studies to impart analgesic effect^8^. A parsimonious interpretation of the findings is that analgesic stimulation acts by increasing GABAergic output from zona incerta to sensory thalamus. Importantly, we also show that the effects of stimulation appear specific to heat pain; perception of non-painful warm stimulation and mechanical pain thresholds were not altered by DBS. However, zona incerta is known to project widely across the brain^5^, and potential relevant off-target effects were not investigated in this study, nor were other pain modalities.

There are differences in findings between this study and previous rodent studies. Most notably, this study did not identify any significant effects of zona incerta DBS on mechanical pain thresholds, while hind paw withdrawal thresholds were seen to increase in rodent models of neuropathic pain^7, 8, 10^. Although unexpected, this may arise from a variety of differences between our study and those performed in rodent models. Primarily, pain in our patients did not arise from clinically relevant sources of neuropathic pain. Additionally, Parkinson disease is known to cause a broad but inconsistent and poorly understood constellation of sensory abnormalities^21^, introducing an important confounder. Performing this study in humans, however, allowed for the first experiment to directly assess the effects of zona incerta stimulation on perceived pain intensities, rather than noxious withdrawal thresholds. Other human studies describing the sensory effects of nearby subthalamic stimulation differ on whether mechanical pain thresholds are modified by stimulation^17, 18^. However, the mechanistic pathway of these effects may also be distinct from that of DBS at zona incerta^22^.

Critically, interpretation of these findings must consider that stimulation was delivered by leads placed in dorsolateral subthalamic nucleus, identified electrophysiologically in this study. In contrast, the portions of zona incerta connected to the spinothalamic tract and sensory thalamus are found in ventral zona incerta, situated medially^5^. As zona incerta is not visualized on 3 T MRI, atlas co-registration was used to approximate contact locations in a common space. These coordinates did not appear to exhibit a gross relationship with analgesic effect. Automated atlas-based lead localization, however, demonstrated much higher variability than imposed by the 2-mm margin of electrophysiological targeting, indicating that this analysis is limited in accuracy, possibly due to limited capture of individual anatomical variance in atlases^23^. Nevertheless, these observations nevertheless suggest that the variations in analgesic effect observed across subjects may be multifactorial in nature, requiring further investigation.

Despite this limitation in study design, stimulation of zona incerta elicited a statistically significant and reproducible effect on perceived heat pain, identifying zona incerta as a strong candidate for neuromodulation of pain. Although clinical translation of this intervention requires substantial additional work, these findings provide a compelling explanation of how subthalamic DBS modulates pain perception in Parkinson patients and present clear avenues for optimization. Foremost, explicit targeting of ventral zona incerta, now directly visualizable in humans^24^, has potential to markedly improve both consistency and magnitude of the analgesic effect. Our finding that stimulation at physiological frequencies is effective also motivates further investigation of other low frequency stimulation paradigms and physiologically inspired patterns. More immediately, these results can be used to inform programming for the large population of subthalamic DBS patients presenting with pain. As we advance our understanding of zona incerta, further research in this direction is warranted, particularly to examine effects on clinically relevant etiologies of pain and sustainability of effects over longer time periods.

## Methods

### Subjects

Outpatient experiments were performed with 9 male and 3 female patients previously implanted with subthalamic DBS leads for treatment of Parkinson disease at the study institution. Patient selection criteria for subthalamic DBS at the institution have been described previously^23, 25^. All patients were implanted with Medtronic (Dublin, Ireland) DBS leads, model 3389, with guidance by 3 T magnetic resonance imaging (MRI), stereotactic navigation, and microelectrode recording. Subjects were implanted at least six months prior to the study and had stable, effective programming parameters. Prior to study, subjects were screened for depression using the Geriatric Depression Scale Short Form^26^. The study was approved by the Institutional Review Boards of the University of Michigan Medical School, experiments were performed in accordance with institutional guidelines and national regulations, and all participants ably provided individual informed consent.

### DBS lead placement

DBS targets were initially assigned from indirect targeting (12 mm lateral, 3 mm posterior, and 4 mm inferior to the mid-commissural point) and adjusted with direct visualization of the ventral border of subthalamic nucleus on 3 T MRI (Philips Achieva 3 T; Philips, Amsterdam, Netherlands). Microelectrode signals were recorded with a Neuroprobe amplified by a Neuro Omega system (Alpha Omega, Alpharetta, GA). Recordings were performed from 15 mm above to 5 mm below the planned target. An experienced electrophysiologist identified the location subthalamic nucleus during surgery. DBS leads were inserted with the tip near the electrophysiologically defined ventral border of subthalamic nucleus. Pulse generators were implanted and connected to DBS leads within 14 days of lead implantation. High-resolution computed tomography scans (GE HD750; General Electric, Boston, MA) were acquired two to four weeks after surgery to verify lead locations.

### Atlas-based estimation of stimulation sites

Stimulation sites were approximated in a common atlas space using Lead-DBS v2.5.2^27^. Post-operative computed tomography scans were co-registered with pre-operative magnetic resonance images using BRAINSFit^28^ or Advanced Normalization Tools^29^. Patient images were then normalized to the MNI ICBM 2009b NLIN ASYM space via Advanced Normalization Tools^29^. Leads were localized within Lead-DBS, with left-sided implants inverted to the right side for analysis. Three subjects were excluded from this analysis due to unavailability of sufficiently high-quality pre- or post-operative imaging. Average site of stimulation was calculated as the arithmetic mean of coordinates of all active contacts for which spatial data was available.

### Deep brain stimulation

**Figure 5:**
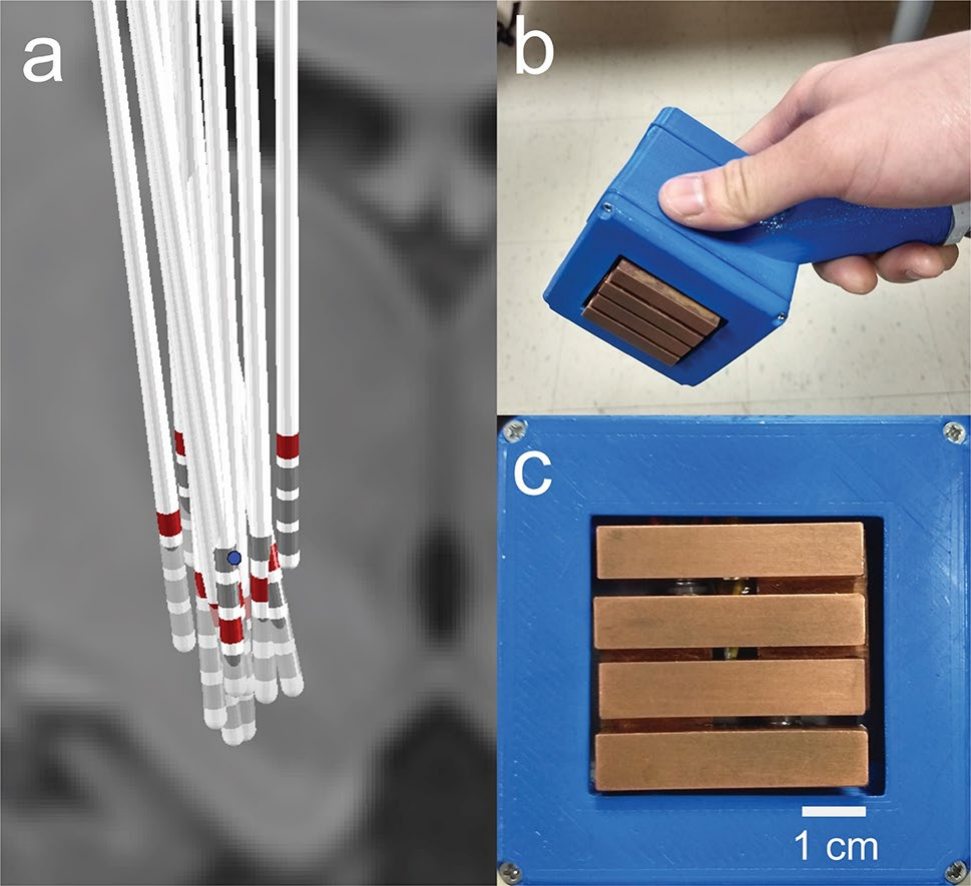
Deep brain stimulation sites and thermal stimulation device. (**a**) Estimated lead locations normalized to MNI space juxtaposed against the MNI152 NLIN 2009b T1 atlas. Active contacts used in the study are indicated in red. The average active contact location is indicated with the blue circle. (**b**) Device used to produce thermal stimuli. (**c**) Detail of device contact surface.

Neuromodulation of zona incerta was achieved using the implanted DBS leads and pulse generators by delivering stimulation to the DBS contact closest to 1.5 mm above the dorsal border of electrophysiological subthalamic nucleus. This results in a 2-mm margin of dorsoventral variability in active contact location across implants, equal to the electrode spacing on model 3389 DBS leads. Three different stimulation frequencies were used: 20 Hz, 60 Hz, and 130 Hz; which reflect the frequency of observed human ZI activity^30^, the frequency of analgesic ZI stimulation in rats^7^, and the frequency of conventional subthalamic DBS stimulation, respectively. Stimulation was delivered contralateral to the side of sensory testing with 60 µs charge-balanced pulses and voltage at 0.5 V below sensory threshold at 130 Hz, with a maximum of 2.0 V. Stimulation settings were set by an experienced clinician using a clinical programmer, with subject and experimenter blinded to stimulation settings. Estimated sites of stimulation in MNI space are shown in Fig. 5a. The average site of stimulation was (11.8, − 11.5, − 1.9) in MNI space.

### Thermal stimulation

A custom device was used to provide thermal stimuli. The contact surface is composed of four parallel copper bars (9 × 10 mm surface), shown in Fig. 5b,c, with temperature controlled by Peltier devices. Two distinct stimuli were produced with the device: nonpainful *Warm* stimuli were produced by setting bars to 39 °C; painful *Hot* stimuli were achieved by setting bars to 45 °C^18^, or the highest temperature tolerable by the subject (always greater than 41 °C).

Thermal stimuli were applied to three sites along the volar forearm: proximal aspect, midpoint, and distal aspect, centered along midline. Each thermal stimulus was tested once at each site for each DBS setting. Application of thermal stimuli followed the sequence listed above with at least 30 s of rest between successive applications. After application of each thermal stimulus, patients were asked to separately rate the intensity (see Supplementary Fig. S1) and evoked pain level of each thermal stimulus on a 10-point scale, with 0 signifying “no sensation/pain” and 10 signifying the “most intense/painful sensation imaginable.” In total, the modulatory effect of each DBS setting on a given thermal stimulation modality was measured using 9–12 independent pain and intensity ratings.

### Mechanical stimulation

Algometry was performed using an Algometer type II device (SBMEDIC Electronics, Solna, Sweden) upon the belly of the extensor digitorum muscle. Three measurements of pressure pain threshold were performed for each test case at locations roughly 1 cm apart. Measurements across test cases were performed at overlapping but non-identical locations.

### Experiment design

#### Exploratory experiments

**Figure 6:**
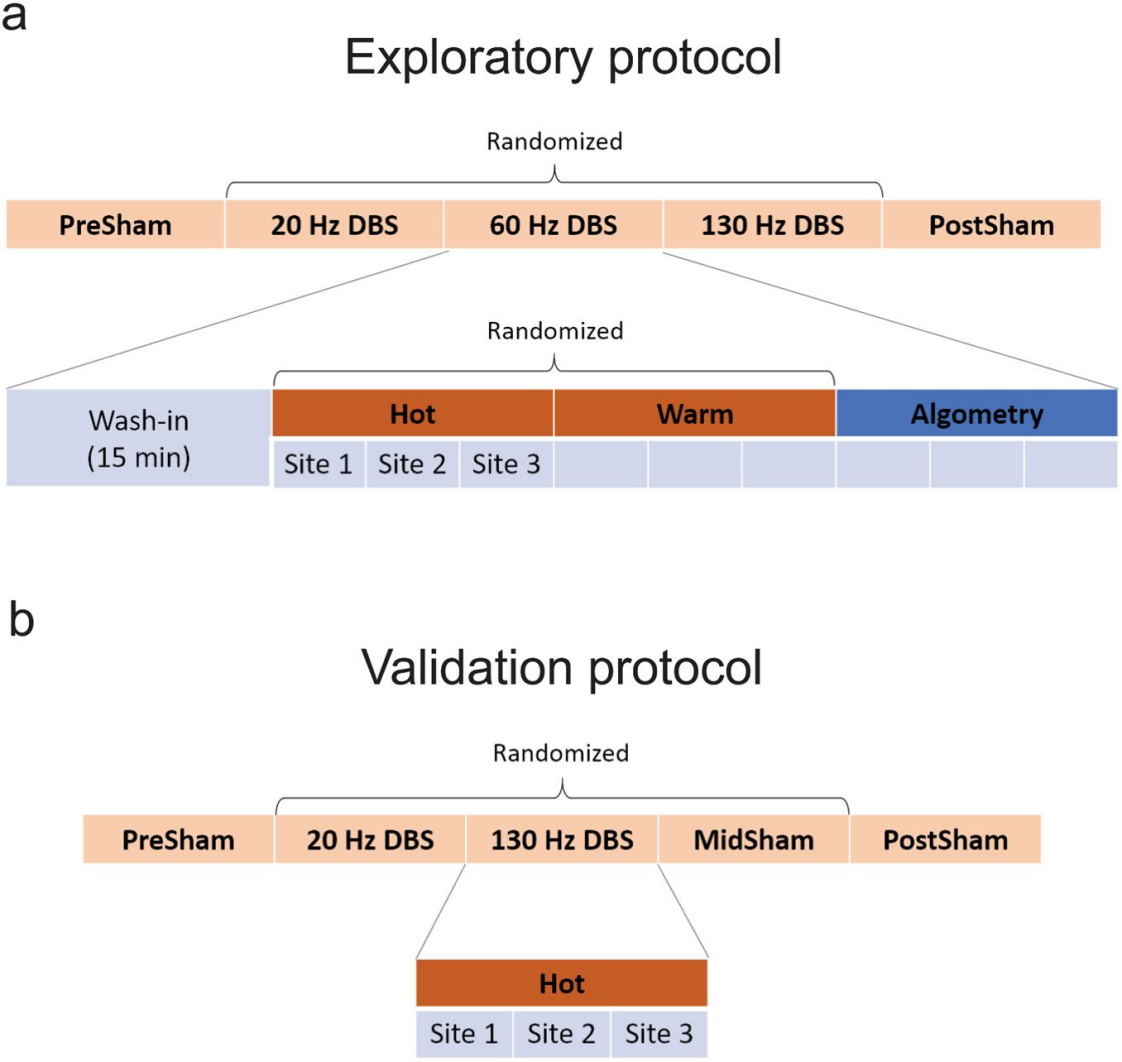
Experimental protocols. (**a**) Protocol for initial exploratory trials. (**b**) Protocol for validation trials including additional sham trial. Order of randomized DBS settings were hidden from both subject and experimenter. Order of randomized thermal stimuli were hidden from subject.

A set of experiments evaluating the effects of 20, 60, and 130 Hz zona incerta stimulation on perceptions of warm, hot, and mechanical stimulation was performed bilaterally with seven outpatient subjects (Fig. 6a).

Each DBS setting was applied for 15 min. The first ten minutes involved no sensory testing to allow for wash-in of potential slow-acting effects. The last five minutes were used to perform sensory testing. Thermal stimuli (warm, hot) were tested in randomized order, counterbalanced across DBS settings. Algometry was performed after thermal testing. Patients were tested unilaterally on one side first, then the other. The first and last DBS settings were sham stimulation. The order of 20, 60, and 130 Hz stimulation was randomized. The experimenter was blinded to stimulation frequency. Test subjects were blinded to stimulation setting (sham and frequency) and thermal stimulation paradigm.

#### Validation experiments

A second set of experiments was performed on an independent cohort of five subjects to specifically validate the effects of 20 and 130 Hz zona incerta stimulation on perceived heat pain (Fig. 6b).

Validation experiments followed a similar protocol to that of exploratory experiments, described above. However, no wash-in time was provided. Instead, thermal stimulation followed immediately (within 5 min) after application of DBS settings. Only hot stimuli were used for sensory testing. These time-saving modifications were motivated by limitations in subject alertness, which was found to decrease significantly when total testing time exceeded three hours. In addition, 60 Hz stimulation was replaced with another period of sham stimulation for three of the five subjects. This third sham trial was introduced to control for potential habituation occurring after first-trial exposure to thermal stimuli. Subjects remained blinded to all deep brain stimulation and sensory stimuli settings. Experimenter was blinded to order of stimulation parameters (including the additional sham period).

### Statistical analysis

A mixed linear model controlling for differences in patient baselines ($\beta_{subject}$) and habituation over time ($\beta_{habit}$) was used to determine the mean effect and significance of each intervention by zona incerta stimulation ($\beta_{DBS}$).

$$VAS = \beta_{subject} + \beta_{habit} trial\,Number + \beta_{DBS} DBS.$$

Each implant was treated as an individual subject during statistical analysis. As such, results obtained from contralateral sides of bilaterally tested subjects were assumed to be independent and have different baselines. Sham stimulation trials were shared across interventions. Results of interest were validated with an independent set of subjects, in lieu of adjusting for multiple comparisons.

## Supplementary Information

Supplementary Information.

## Data Availability

Data from this study are available upon reasonable request to the corresponding author.
