# Supplementary material for: Stimulation of zona incerta selectively modulates pain in humans
Source: Sci Rep. 2021 Apr 26;11:8924. doi: 10.1038/s41598-021-87873-w (PMC8076305; doi:10.1038/s41598-021-87873-w)
Supplement: Supplementary file 1 — Supplementary Information. [file 41598_2021_87873_MOESM1_ESM.docx]

Stimulation of zona incerta selectively modulates pain in humans

Charles W Lu^1,2^, Daniel E Harper^3^, Asra Askari^1^, Matthew S Willsey^1,2^, Philip P Vu^1,2^, Andrew D Schrepf^4^, Steven E Harte^4,5^, Parag G Patil^1,2,*^

^1^ Department of Neurosurgery, University of Michigan,1500 E Medical Center Drive, SPC 5338 Ann Arbor MI 48109-5338 USA
^2^ Department of Biomedical Engineering, University of Michigan, Ann Arbor, MI USA
^3^ Department of Anesthesiology, Emory University, Atlanta, GA USA
^4^ Department of Anesthesiology, Chronic Pain and Fatigue Research Center, University of Michigan, Ann Arbor, MI USA
^5^ Division of Rheumatology, Department of Internal Medicine, University of Michigan, Ann Arbor, MI USA
^*^ corresponding author

Parag G Patil
UH South, Room F2306
1500 E Medical Center Drive, SPC 5338
Ann Arbor, MI 48109
(734) 936-9579
pgpatil@med.umich.edu

# Supplement


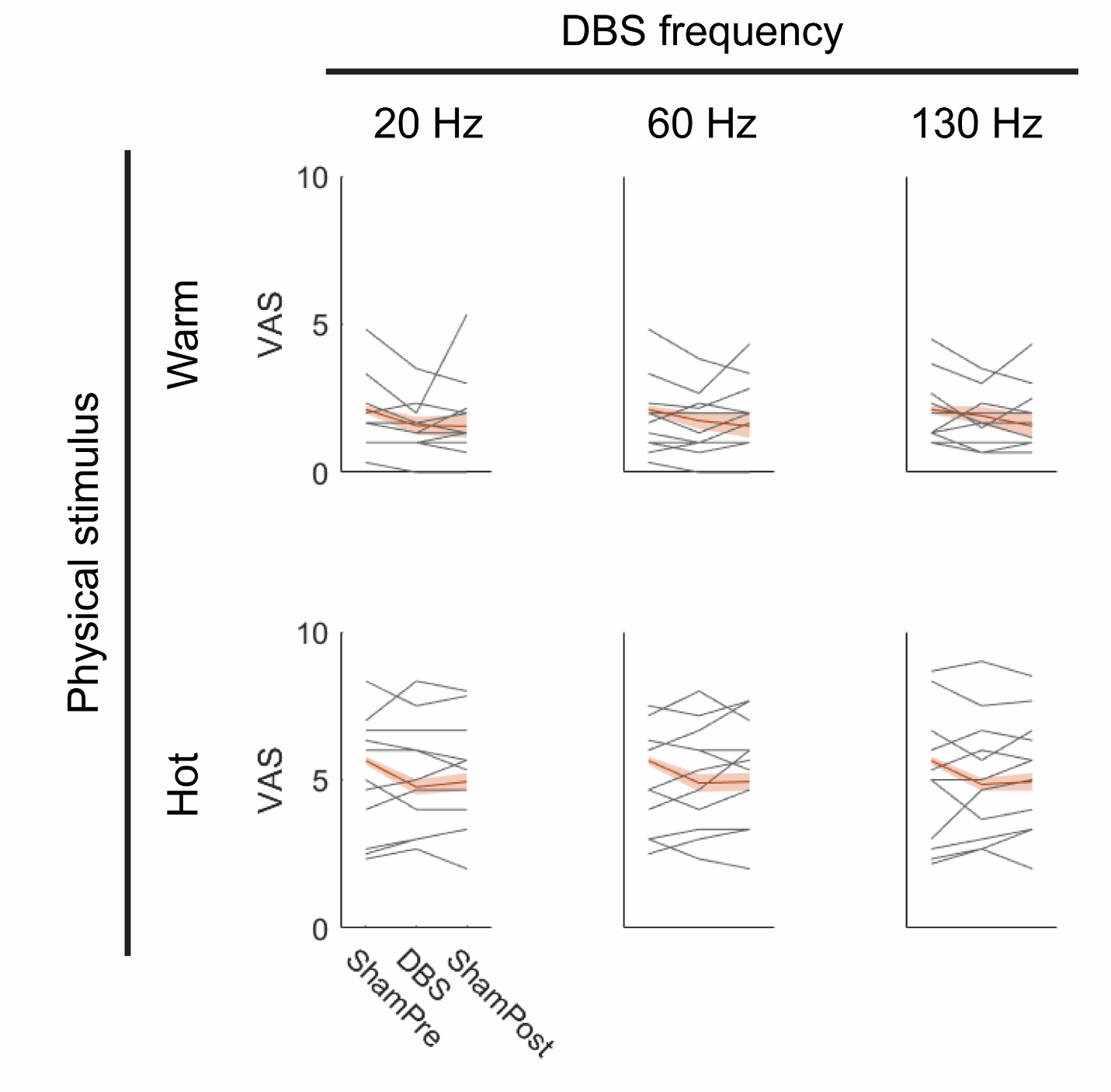


SUPP FIGURE 1: Effects of DBS on perceived intensity from warm and hot stimuli. Gray lines show mean intensity scores across arm sites for each subject-implant. Red lines show average across implants with standard error of the mean shaded. *n* = 99 trials for all plots, with sham trials shared across DBS frequencies.


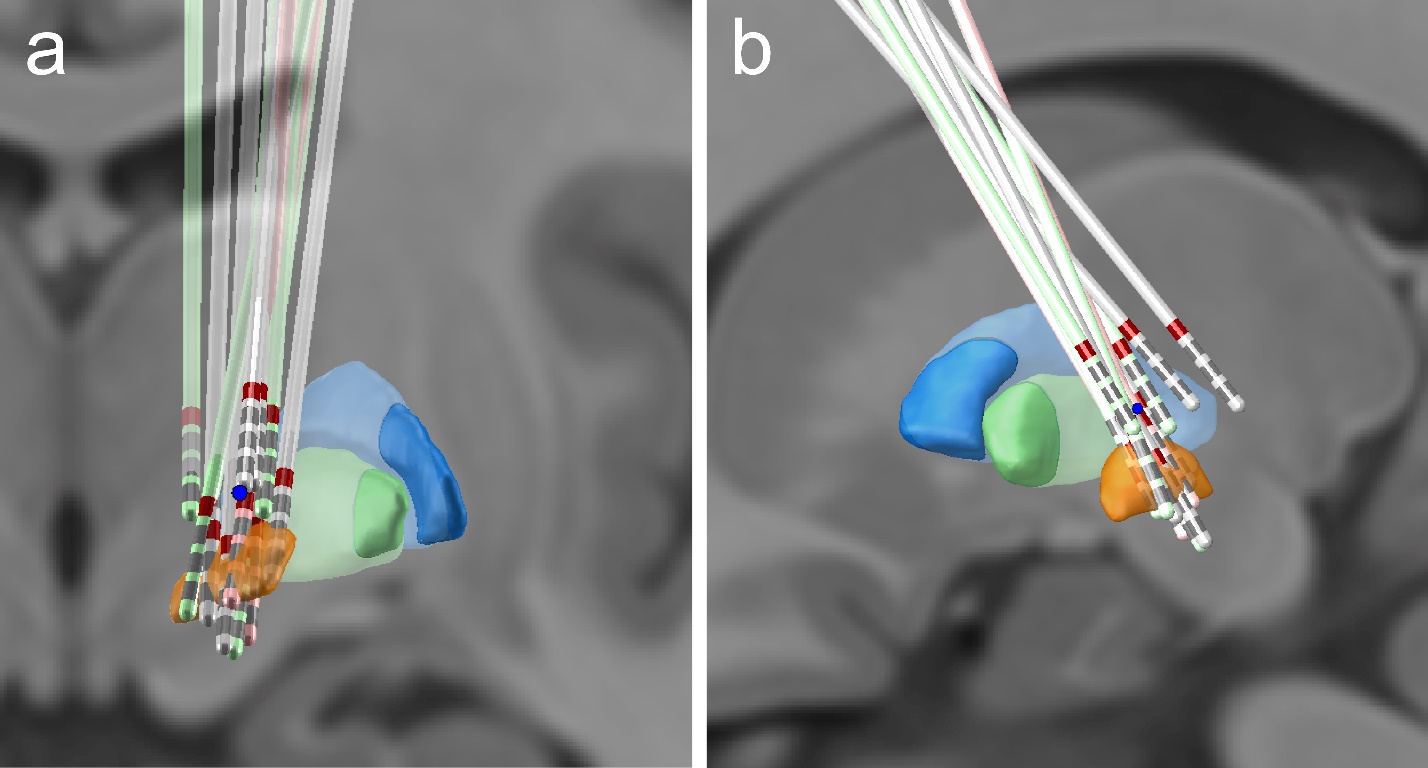
SUPP FIGURE 2: Locations of responder and non-responder stimulation sites juxtaposed against the MNI152 NLIN 2009b T1 atlas, in a) coronal and b) sagittal views. Active contacts used in the study are indicated in red. The average active contact location is indicated with the blue circle. Green leads indicate pain reduction of 15% or more; gray, 0 to 10% reduction in pain; and red, pain augmentation. Boundaries of the STN (orange), globus pallidus interna (green), and globus pallidus externa (blue) in MNI space are shown.
